# Accessibility, Relevance, and Impact of a Symptom Monitoring Tool for Home Hospice Care: Theory Elaboration and Qualitative Assessment

**DOI:** 10.2196/51789

**Published:** 2024-05-23

**Authors:** Karla T Washington, Debra Parker Oliver, Allison K Donehower, Patrick White, Jacquelyn J Benson, Patrick G Lyons, George Demiris

**Affiliations:** 1 Department of Medicine School of Medicine Washington University in St. Louis St. Louis, MO United States; 2 Goldfarb School of Nursing Barnes-Jewish College St. Louis, MO United States; 3 Department of Human Development and Family Science College of Education and Human Development University of Missouri Columbia, MO United States; 4 BJC Hospice St. Louis, MO United States; 5 Department of Medicine School of Medicine Oregon Health & Science University Portland, OR United States; 6 Department of Medical Informatics and Clinical Epidemiology School of Medicine Oregon Health & Science University Portland, OR United States; 7 Department of Biostatistics, Epidemiology, & Informatics School of Medicine University of Pennsylvania Philadelphia, PA United States; 8 Department of Biobehavioral Health Sciences School of Nursing University of Pennsylvania Philadelphia, PA United States

**Keywords:** caregivers, home care services, hospice care, signs and symptoms, technology, mobile phone

## Abstract

**Background:**

Early users found Engagement and Visualization to Improve Symptoms in Oncology Care (ENVISION), a web-based application designed to improve home management of hospice patients’ symptoms and support patients’ and family caregivers’ well-being, to be generally useful and easy to use. However, they also raised concerns about potential challenges users with limited technological proficiency might experience.

**Objective:**

We sought to concurrently accomplish two interrelated study aims: (1) to develop a conceptual framework of digital inclusivity for health information systems and (2) to apply the framework in evaluating the digital inclusivity of the ENVISION application.

**Methods:**

We engaged ENVISION users (N=34) in a qualitative study in which data were collected via direct observation, think-aloud techniques, and responses to open-ended queries. Data were analyzed via theory elaboration and basic qualitative description.

**Results:**

Accessibility, relevance, and impact were identified as 3 essential considerations in evaluating a health system’s digital inclusivity. Study findings generally supported ENVISION’s digital inclusivity, particularly concerning its perceived relevance to the work of family caregivers and hospice clinicians and its potentially positive impact on symptom management and quality of life. Limitations to ENVISION’s digital inclusivity centered around issues of accessibility, particularly operability among individuals with limited technological knowledge and skills.

**Conclusions:**

The Accessibility, Relevance, and Impact conceptual framework of digital inclusivity for health information systems can help identify opportunities to strengthen the digital inclusivity of tools, such as ENVISION, intended for use by a broad and diverse range of users.

## Introduction

### Background

Hospice is a health care delivery model and a philosophy of care focused on reducing pain and promoting quality of life among patients who are terminally ill and their families [1]. In the United States, hospice care is most often provided in patients’ homes [2]. While more intensive staffing is available during acute medical crises, routine home hospice care consists of only periodic visits from nurses, nursing aides, social workers, chaplains, and others operating under the direction of a hospice physician [3]. A total of 3- to 4-hour-long weekly home visits may be typical for an established patient. Thus, responsibility for the overwhelming majority of home hospice care falls to patients’ family members and friends (referred to as *family caregivers*), who are typically unpaid and often lack formal health care training [4-6].

Hospice family caregivers are commonly tasked with in-home management of patients’ symptoms, including pain, shortness of breath, anxiety, and fatigue. Recent population-based research indicates that >78% of family caregivers who assist with symptom management in the last month of a patient’s life report difficulty doing so [7]. These findings are consistent with those of numerous other studies highlighting the reality that symptom management challenges are a significant source of stress for many hospice family caregivers [8-11]. These challenges, coupled with lack of a standardized processes for real-time symptom reporting and monitoring in home hospice care, commonly result in suboptimal home management of patients’ symptoms [12].

### Engagement and Visualization to Improve Symptoms in Oncology Care

**Figure 1:**
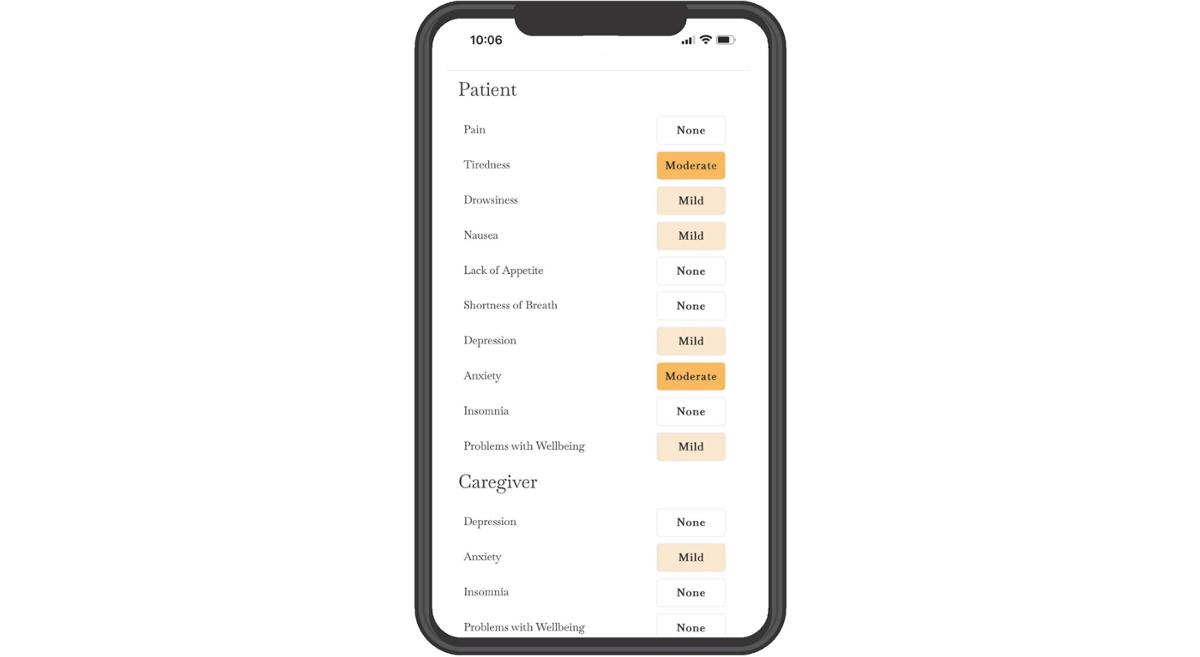
Mobile version of the application Engagement and Visualization to Improve Symptoms in Oncology Care (ENVISION), showing a sample patient and caregiver scorecard.

**Figure 2:**
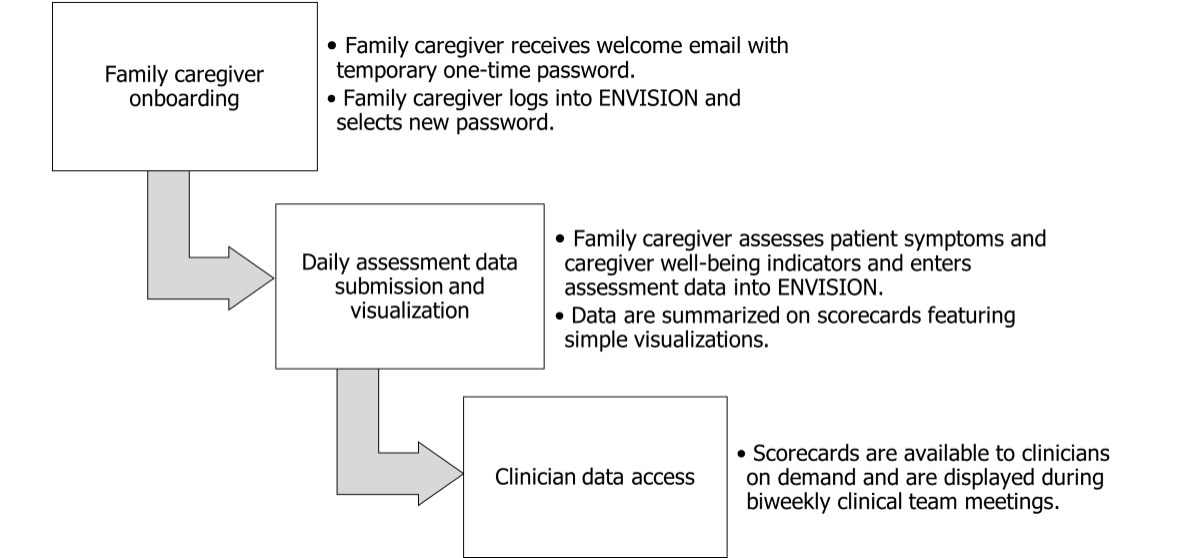
Engagement and Visualization to Improve Symptoms in Oncology Care (ENVISION) workflow.

Engagement and Visualization to Improve Symptoms in Oncology Care (ENVISION) is a secure, web-based application designed to improve home management of hospice patients’ symptoms and support patients’ and family caregivers’ well-being by improving the exchange of information between family caregivers and hospice clinicians [13]. It uses daily symptom and well-being data entered on the internet by family caregivers to create simple visualizations summarized in a patient and caregiver scorecard (Figure 1), allowing hospice clinicians to quickly identify areas of concern. These scorecards are displayed during biweekly hospice interdisciplinary team meetings and are available on demand to hospice clinicians outside of regularly scheduled meetings. A workflow diagram illustrating ENVISION’s use is provided in Figure 2. Optional views, including longitudinal graphs of individual or combined symptoms, are also available to clinician users. Although ENVISION was initially developed specifically for advanced cancer care, its use has been expanded to include care for hospice-eligible individuals experiencing any life-limiting illness.

### Digital Inclusivity

ENVISION was created over several years with significant involvement of hospice family caregivers, clinicians, and administrators. While early research broadly supported ENVISION’s usefulness and ease of use, it also raised concerns about potential barriers to use that might be experienced by family caregivers with limited technological skills or resources [13]. These concerns echo those voiced as part of an ongoing discussion in health care regarding digital inclusivity [14], broadly defined as “the ability of individuals and groups to access and use information and communication technologies [15].”

Digital inclusivity, particularly as it pertains to digital health technologies, is a salient concern on multiple social levels [16]. Individually, users vary with regard to their level of digital literacy and their ability to personally take advantage of available technological resources [17]. For example, an individual may struggle to discern differences between trustworthy and untrustworthy sources of health information, may have functional limitations (for example, vision or hearing impairment), or may be unable to afford home internet access. Similarly, families and other social groups differ in their degree of collective technological resources, a reality evident when less technologically equipped individuals benefit from the digital knowledge and skills of family members and friends. A family member assisting a patient in accessing their health care portal would be one example [18]. Communities can also be considered more or less digitally inclusive based on the adequacy of the infrastructure (such as home broadband connectivity or public Wi-Fi networks) available to meet residents’ technological needs [15].

### Study Aims

Our initial aim in conducting the study described herein was to better understand early ENVISION users’ concerns regarding the application’s digital inclusivity. However, in planning our study, we struggled to identify an existing conceptual framework to guide our research, given our plan to explore digital inclusivity as a quality of an individual application (rather than, for example, a community). Thus, we added a second study aim: to engage ENVISION users in a process of theory elaboration, resulting in a conceptual framework of digital inclusivity for health information systems. In this way, we sought to inform future ENVISION enhancements while contributing to the broader emerging science of digital inclusivity in health care. Thus, our finalized study aims were as follows: (1) to develop a conceptual framework of digital inclusivity for health information systems and (2) to apply the framework in evaluating the digital inclusivity of the ENVISION application.

## Methods

### Setting, Participants, and Recruitment

As part of ENVISION’s ongoing, iterative, user-centered design [19], we recruited hospice family caregivers and clinicians (nurses, physicians, social workers, and chaplains) to participate in a qualitative research study [20]. We partnered with the university’s Institute of Clinical and Translational Sciences’ Recruitment Enhancement Core to recruit hospice family caregivers via flyers, targeted email blasts, social media posts, and listing of the study on a public-facing website. Family caregivers were eligible for inclusion if they were aged ≥18, able to speak and read English, and current or former (within the prior year) family caregivers of a patient receiving services from a Medicare-certified US hospice agency. We recruited hospice clinicians via social media posts and email blasts from professional hospice organizations, targeted emails to prior research partners, and presentation of the study opportunity at scheduled meetings of hospice agencies with which we had established partnerships. Hospice clinicians were eligible for study inclusion if they were aged ≥18, able to speak and read English, and currently employed or affiliated with a Medicare-certified US hospice agency.

### Data Collection

All consenting participants met online individually with a research team member for approximately 30 to 45 minutes via a university-managed, Health Insurance Portability and Accountability Act–compliant Zoom (Zoom Video Communications) account [21]. At the start of each call, the researcher provided assistance in using Zoom’s screen share feature, which the researcher later used to observe the participants completing a series of structured tasks in the ENVISION application. In addition, the researcher provided instruction in the think-aloud technique [22,23], explaining that the participants would be asked to verbalize their thinking as they navigated the application and completed specific tasks. The researcher also informed the participants that they would be asked to answer a series of open-ended questions about their perceptions of the application and its potential use in hospice care. Finally, the researcher confirmed the participants’ understanding and began recording the session with the participants’ knowledge and permission.

### Structured Tasks

During the recorded Zoom session, family caregivers were sent a personalized email with a brief welcome message, a link to the ENVISION website, and a temporary one-time password (an automatically generated alphanumeric string of characters). As their first observed task, family caregivers were asked to navigate to the ENVISION website, enter the site using their email address and one-time password, and choose a new password. They were then asked to recall a typical caregiving day and enter corresponding symptom and well-being data for the patient and themselves into the ENVISION application. Next, they were asked to navigate to the patient and caregiver scorecard, which summarized the data they had just entered, and answer questions that required them to interpret simple data visualizations (labeled rectangles filled with different shades of orange ranging from none or white to dark or bright orange to reflect greater symptom intensity). Finally, they were asked to exit the application. After completing these structured tasks, they were asked a series of open-ended questions, including, for example, “What made it easy to use ENVISION?” “What made it challenging?” and “Which symptom(s) would be most important to communicate to the hospice team? Why?”

Hospice clinicians were also observed navigating to the ENVISION website, entering the site using their email address and temporary password, and selecting a new password. Because the researcher had entered them into the system as a clinician when generating their welcome email, clinicians were taken to a screen that included a list of fictitious patients’ names and medical record numbers upon logging into the system. When they reached this screen, clinicians were asked to navigate to a specific patient’s information (this required them to locate and click on the patient’s name, but they were not given these specific instructions). Clicking on the patient’s name took them to a screen that included a daily patient and caregiver scorecard that featured visualizations similar to those shown to family caregivers. This page also included a simple line graph that allowed clinician users to view the intensity of one or more symptoms or well-being indicators over time by clicking a box next to the appropriate symptoms or indicators (users were not provided with these specific instructions). While on this page, clinicians were asked questions that required them to interpret the colored boxes on the patient and caregiver scorecard; select and deselect specific symptoms on the longitudinal graph; and interpret trends, including symptom co-occurrence over time, shown via the graphed data. Finally, they were asked to exit the system and answer a series of open-ended questions, including, for example, “How, if at all, would having [information provided via ENVISION] affect how you do your job?” and “Describe how you would access ENVISION. For example, would you use a desktop computer, tablet, or smartphone? Would you use the application from the hospice agency office, patients’ homes, or elsewhere?”

### Data Preparation

In preparation for data analysis, we contracted with a third-party service to transcribe audio files of participants’ recorded Zoom sessions verbatim. We then imported the resulting transcripts into NVivo qualitative analysis software (Lumivero). Complete copies of all audio and video files of participants’ recorded Zoom sessions and corresponding field notes were stored in a secure Box folder made available to all institutional review board–approved research team members throughout data analysis.

### Data Analysis

Our analysis was broadly informed by the work of organizational management researchers Fisher and Aguinis [24], who described a process they referred to as theory elaboration, defined as “the process of conceptualizing and executing empirical research using preexisting conceptual ideas or a preliminary model as a basis for developing new theoretical insights by contrasting, specifying, or structuring theoretical constructs and relations to account for and explain empirical observations.” As part of this process, we engaged in vertical contrasting, which entailed adapting an existing conceptual framework (described in detail in the next paragraph) developed for one level of analysis to examine a phenomenon at another level. In doing so, we sought to determine which aspects of the framework functioned similarly on both levels of analysis and which functioned differently. We also engaged in construct specification, seeking to refine the constructs articulated in the original framework and to introduce new constructs when the existing constructs failed to capture important aspects of the phenomenon under investigation (in our case, ENVISION’s digital inclusivity). At times, this involved construct splitting, a process whereby we split existing constructs into more specific dimensions if more conceptual specificity was needed to capture important aspects of ENVISION’s digital inclusivity. Finally, we engaged in structuring, or identifying relationships between and among constructs, remaining open to new relation structures.

To accomplish these analytic activities (ie, contrasting, specifying, and structuring), 2 researchers (KTW and AKD) first reviewed all study transcripts, video files, and field notes. They then met to develop an initial codebook based on the elements of an existing framework: *Building Digital Communities: A Framework for Action* [15], created by the Institute of Museum and Library Services, the University of Washington Technology and Social Change Group, and the International City or County Management Association. As its name suggests, this framework was created to promote digital inclusivity at the community level. Consistent with this purpose, it articulated 13 principles for community-wide digital inclusivity, including access principles (which addressed community infrastructure needs), adoption principles (which focused on community members’ facilitators and barriers to use), and application principles (which specified areas where deployment of digital technologies would be likely to enhance community members’ lives). The original framework’s principles and corresponding definitions are provided in Multimedia Appendix 1.

We originally envisioned development of the initial codebook as a relatively straightforward process in which most, if not all, constructs articulated in the original framework would be initially retained and then refined in subsequent analytic steps. However, it soon became apparent that some of the original principles had limited applicability in the context of an individual application and should, therefore, be de-emphasized or excluded in the early stages of our analysis. For example, the application principles outlined in the original framework identified specific community sectors, such as education and public safety, where the deployment of technologies was deemed likely to benefit community well-being. However, digital health tools are, by definition, intended for deployment in health care (and, in the case of ENVISION, more specifically in hospice care). Thus, we omitted them from the initial codebook, feeling confident they would neither enrich our understanding of ENVISION’s digital inclusivity nor ultimately represent constructs comprising our adapted conceptual framework.

After completing the initial codebook, KTW and AKD independently coded approximately 15% of the study transcripts, consulting video recordings and field notes as needed for context or clarification of transcribed data. KTW and AKD then met to make substantive modifications to the codebook to enhance its goodness of fit with the data. Examples of changes made at this stage included specifying that *affordability* referred to ENVISION’s initial and ongoing costs and should, thus, be relabeled as *affordability and sustainability* (construct specification) and dividing *design for inclusion* into *perceivability*, *operability*, and *comprehensibility* (construct splitting), as the available data suggested that these were conceptually meaningful distinctions. We then used the modified codebook to code the entire data set, meeting afterward to compare individual coding decisions (resolving discrepancies via discussion and arriving at consensus), finalize our code definitions, and group related codes into broader categories that comprised our resulting conceptual framework and shed light on ENVISION’s strengths and limitations with regard to digital inclusivity.

### Ethical Considerations

All research activities were reviewed and approved by the Washington University in St Louis Institutional Review Board (#202105172).

Individuals interested in study participation were provided with contact information for our study coordinator, who screened potential participants for eligibility, obtained verbal informed consent for those interested in participating, and coordinated all subsequent research activities including participant payments of US $40 sent via check to the mailing address of the participants’ choice.

## Results

### Overview

A total of 34 individuals participated in our qualitative research study, enabling the concurrent achievement of 2 interrelated study aims: (1) to develop a conceptual framework of digital inclusivity for health information systems and (2) to evaluate the digital inclusivity of the ENVISION application (participant characteristics are summarized in Table 1). In the following sections, we present our study findings, beginning with a brief overview of our conceptual framework and its essential elements. We then provide an in-depth description of the framework, illustrating its specific constructs with examples from our evaluation of ENVISION’s digital inclusivity.

**Table 1:** Summary of participant characteristics (N=34).

Characteristic			Family caregivers (n=10), n (%)		Hospice clinicians (n=24), n (%)
**Age range (y)**					
	18-29	1 (10)		2 (8)	
	30-39	1 (10)		5 (21)	
	40-49	1 (10)		6 (25)	
	50-59	2 (20)		8 (33)	
	60-69	3 (30)		3 (13)	
	≥70	2 (20)		0 (0)	
**Gender**					
	Man	1 (10)		5 (21)	
	Woman	9 (90)		19 (79)	
**Race**					
	Black	3 (30)		0 (0)	
	White	7 (70)		24 (100)	
**Ethnicity**					
	Hispanic	0 (0)		1 (4)	
	Non-Hispanic	10 (100)		23 (96)	
**Relationship to patient**					
	Spouse or partner	2 (20)		N/A^a^	
	Adult child	5 (50)		N/A	
	Other	3 (30)		N/A	
**Highest formal education**					
	Some college or trade school	2 (20)		N/A	
	Associate’s degree	2 (20)		N/A	
	Bachelor’s degree	3 (30)		N/A	
	Graduate or professional degree	3 (30)		N/A	
**Profession**					
	Chaplain	N/A		5 (21)	
	Nurse	N/A		7 (29)	
	Other	N/A		1 (4)	
	Physician	N/A		3 (13)	
	Social worker	N/A		8 (33)	
**Professional experience (y)**					
	0-5	N/A		6 (25)	
	6-10	N/A		5 (21)	
	11-15	N/A		4 (17)	
	16-20	N/A		2 (8)	
	21-25	N/A		2 (8)	
	>25	N/A		5 (21)	

^a^N/A: not applicable.

### The Accessibility, Relevance, and Impact Conceptual Framework of Digital Inclusivity for Health Information Systems

**Figure 3:**
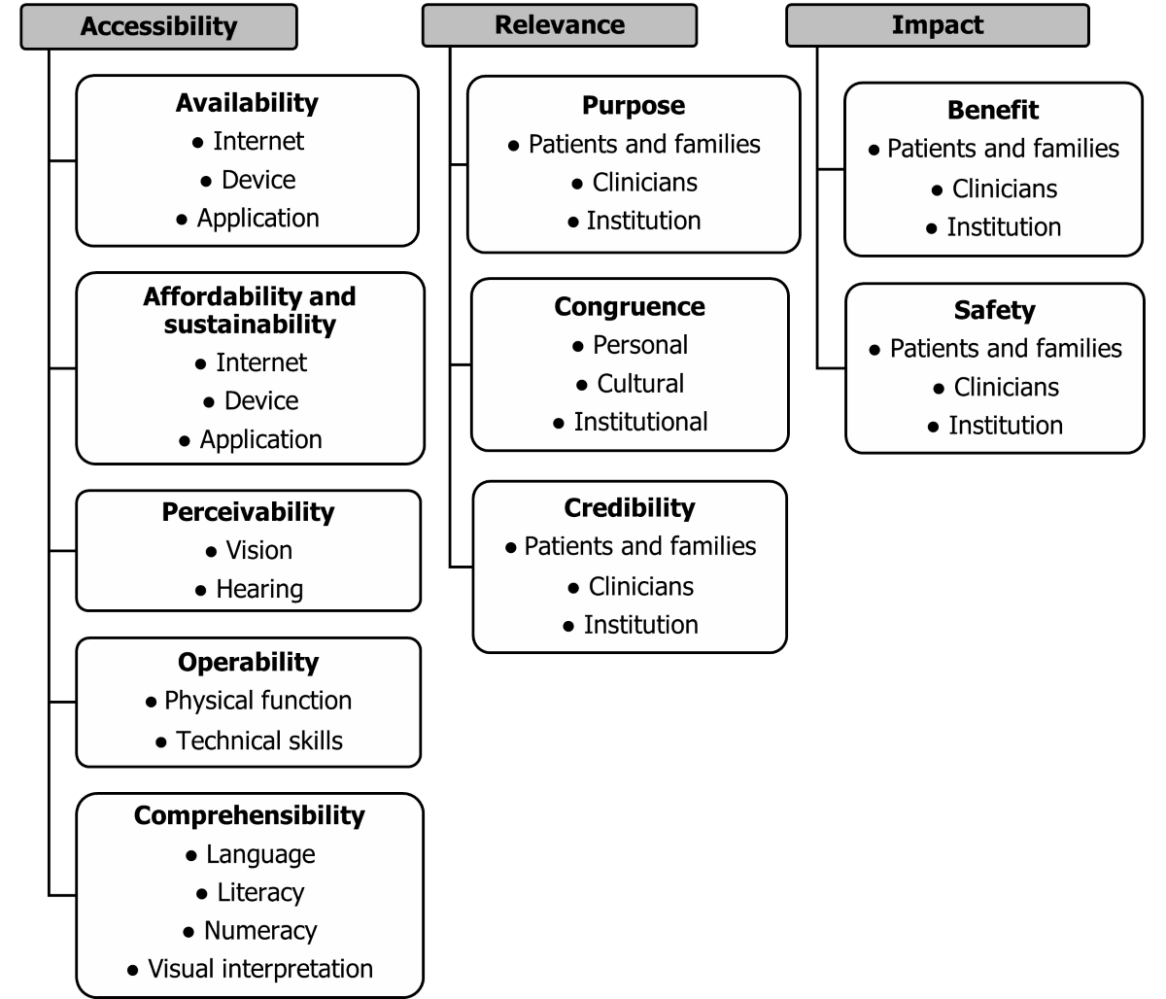
The Accessibility, Relevance, and Impact (ARI) conceptual framework of digital inclusivity for health information systems.

Our analysis resulted in the development of a conceptual framework of digital inclusivity for health information systems that comprises 3 essential elements: accessibility, relevance, and impact (Figure 3). Per the Accessibility, Relevance, and Impact (ARI) framework, in evaluating a health information system’s accessibility, the availability and affordability or sustainability of access to the internet (for web-based applications); necessary devices (eg, computers, smartphones, and tablets); and the application or system itself must be considered. Also relevant are a system’s perceivability (the extent to which it can be used by individuals with different sensory abilities, such as visual or hearing impairments), operability (the extent to which it can be used by individuals with different physical abilities or technological proficiencies), and comprehensibility (the extent to which users can understand and accurately interpret the system’s content). A health information system’s relevance is also key to its digital inclusivity. Digitally inclusive systems are useful (ie, they fulfill a clear purpose); trustworthy (ie, they are viewed as credible); and aligned with users’ values, beliefs, customs, and preferences (ie, they are congruent). Finally, the framework suggests that the evaluation of a health information system’s digital inclusivity requires consideration of its impact, that is, the extent to which it improves (or would be expected to improve) users’ lives (benefit) and the presence or absence of protection from web-based threats (eg, malware and data breaches) associated with the system’s use (safety).

### Evaluating ENVISION’s Digital Inclusivity Using the ARI Framework

#### Accessibility

Data describing ENVISION’s availability referenced the presence or lack of internet access, technological devices, or otherwise referred to potential users’ ability to retrieve the application. Study participants’ feedback and experiences related to availability were generally positive. Family caregivers either did not comment on ENVISION’s availability or were positive in their responses; no family caregivers identified availability-related barriers to accessing the application. Although hospice clinicians also expressed generally positive perceptions of ENVISION’s availability, some clinicians noted potential limitations. For example, one clinician stated that the application would be inaccessible for some, including “people without internet, without computers.” Another explained, “Keep in mind that your patients or family members...may not have access to technology.” With regard to the application’s availability to clinicians, one participant emphasized the importance of integrating any new tools with the existing electronic health record:

Sometimes more tools are better, but sometimes more tools take more time...If this could be embedded into our current [EHR], I could find that helpful. To log out and then log in to something else or to toggle between two applications, I think, would be more cumbersome.

Data describing ENVISION’s affordability and sustainability referenced the cost of ENVISION itself or other resources (eg, internet access and technological devices) required to access and use the application. Participants who commented on ENVISION’s affordability and sustainability expressed generally positive perceptions. Family caregivers, many of whom likely assumed the application would be included in routine hospice care and thus free of charge to patients and families, did not directly raise the issue. Two clinicians (one of whom also occupied an administrative role) noted the importance of making the application affordable and sustainable for hospice agencies, stressing the issue of cost-effectiveness, and linking the application to clinically relevant outcomes. When asked if they would recommend routine use of ENVISION in hospice care, they replied as follows:

I think you’d have to look at...cost, [but] this program will definitely improve outcomes for symptom management.

Data describing ENVISION’s perceivability referenced the extent to which the application could be used by individuals with different sensory abilities, particularly with regard to vision. Participants’ comments regarding the perceivability of ENVISION varied. Some suggested that the font size was too small:

The print would have to be a little larger. I think, in general, anything to do with [older adults] should be larger.

Others stated the opposite, describing ENVISION’s text and images as “not too small.” One participant who did not cut and paste the one-time password from their welcome email into the log-in screen noted that “for people [who] are...visually challenged, [entering] the password [could be] a bit of a headache.” A clinician described ENVISION as a “wonderful option for patients and family members” but suggested that it not be required, as some may not be “able to see and hear...and all those sorts of things.”

Data describing ENVISION’s operability referenced the extent to which it could be used by individuals with different physical abilities or technological proficiencies. Participants provided mixed feedback on ENVISION’s operability. Overall, for family caregivers, logging into the application for the first time (which required them to enter their email address and a one-time password emailed to them during the call) was more challenging than using it once they logged in. One family caregiver stated as follows:

If you’re not a computer-savvy sort of person, [logging in the first time] could be a challenge.

This was particularly the case for users (family caregivers and hospice clinicians) who did not cut and paste their one-time password from their welcome email into the log-in screen:

I have to write [the one-time password] down because I won’t remember it.

Another user provided a specific suggestion related to this issue:

Do you know, with this system, can you instruct people to copy the initial password and paste it in, just to make it easier for people?

After entering the system, however, family caregivers could easily enter symptom and well-being data into ENVISION, describing this process as “pretty simple” and “really easy.” One family caregiver stated, “That took less than 30 seconds,” while another explained as follows:

I think it was really easy. I really liked how there’s a definition [of each symptom or wellbeing indicator]...It’s comparable to other applications I use for work...I think that would be pretty straightforward for the general population, too.

Although this user saw the information symbol (lowercase “i” with a circle around it) located next to each symptom and well-being indicator and knew to click on it for more information, others required prompting before being able to do so. Ultimately, however, 100% of the users who expressed a desire for more information about a symptom or well-being indicator were able to successfully obtain that information by clicking on the information symbol independently or after receiving the following verbal prompt from the researcher: “Is there anything on the screen that might give you more information about that?”

Clinicians’ comments regarding ENVISION’s operability with regard to logging into the system and navigating the application generally mirrored those of family caregivers. They described the overall application as generally operable while emphasizing that it might be challenging for individuals to use if they were physically unable to type or “[could not] even use a smartphone” (the issue of family caregivers’ ability to independently use the application is further described when discussing *congruence* under the *Relevance* section, as numerous clinicians expressed concern that they would be tasked with training and assisting technologically challenged family caregivers with ENVISION’s use, requiring significant amounts of their already limited work time). Most of the unique data about ENVISION’s operability for clinicians focused on using the interactive graphs that enabled longitudinal viewing of symptoms and well-being indicators (these graphs were available only to clinician users). To choose which symptoms or well-being indicators appeared on the longitudinal graphs, clinician users needed to click a box next to the appropriate symptoms or indicators, which multiple users failed to do without prompting or considerable thought. For example, one clinician user’s think-aloud data included the following:

I’m guessing maybe—I was looking at it on my computer—the little check boxes underneath the graph might affect the graph, I guess...Now, let’s see...[begins clicking on boxes and noting changes to the graph].

Another suggested that the application be modified to include “some education on what that graph is and how to utilize it.” Other clinician users appeared to interact with the graph more intuitively and were observed easily manipulating it. One such clinician stated as follows:

I didn’t have any problem with it. I’m middle-aged and...pretty computer-literate. I didn’t have any problems with it at all.

Data describing ENVISION’s comprehensibility referenced the extent to which users could understand and accurately interpret the application’s content. ENVISION’s comprehensibility was determined to be mixed. Overall, family caregivers could easily comprehend ENVISION’s content, successfully entering symptom and well-being indicators and accurately interpreting the data visualizations (labeled boxes shaded in different intensities to reflect symptom and indicator intensity) featured on patient and caregiver scorecards. Several described the content as easy to understand, using words and phrases such as “straightforward” and “written in plain English.” Among family caregivers, comprehensibility challenges were limited to understanding the definition of specific symptoms or well-being indicators; however, most of these challenges were resolved when the users clicked on the information symbol and were shown a definition. Users commonly clicked on the information symbol next to “well-being,” expressing confusion about what it entailed (eg, “Is that mental well-being or is that physical?”). Differentiating between “tiredness” and “drowsiness” was also challenging for numerous family caregivers. Among the comprehensibility challenges that were not resolved by clicking on the information symbol, nonspecificity (eg, uncertainty whether they were being asked to report on generalized anxiety or anxiety specific to the hospice experience and confusion about the insomnia indicator: “Is that insomnia [as in] you can’t sleep, or is it just that you know you have to get up because you have to check [on the patient]?”) was by far the most common. Clinicians recommended that longitudinal graphs be labeled with complete descriptions of symptoms and well-being indicators rather than shortened descriptors (eg, use “shortness of breath” rather than just “breath”). However, this may have been more of a design preference than an issue related to comprehensibility. One family caregiver recommended that features beyond the patient or caregiver name and uploaded photograph be included to remind the family caregiver when they were being asked to report on the patient’s experience or their own:

You could say, “Now we’re...addressing you, not the patient” or however you would want to say it...Make it clearer which page is for the patient and which is for the caregiver.

#### Relevance

Data describing ENVISION’s purpose referenced its perceived usefulness. Most users identified a clear and important purpose for ENVISION in their respective roles. Family caregivers repeatedly emphasized the importance of reporting symptoms and well-being indicators to the hospice team (patient pain was most commonly cited as a high-priority symptom to communicate). Feedback on the importance of the general well-being indicator, however, was mixed among family caregivers. Some family caregivers selected it as the most critical piece of data to communicate, while others saw it as redundant:

I feel that was a culmination of all the options that you gave me to begin with. If I’m already addressing each one of those issues individually…maybe I didn’t necessarily need to rate it separately.

One caregiver was unclear why caregiver insomnia was included as a well-being indicator:

If I had insomnia, how would the healthcare provider help me with that?

With a few exceptions, clinician participants could readily identify a purpose for ENVISION in their clinical role, evident from representative statements as follows:

I think it would help me do my job better due to it being so precise, and I go back to the [patient and caregiver scorecard]. It’s a lot easier for me to see what’s going on with that patient the way that was presented than what I’m doing now in a chart, where I have to click and copy and paste and go here and there and everywhere [to] different notes and things like that.

A chaplain explained how using ENVISION would enhance spiritual care:

[ENVISION might help me decide] how soon I might want to make another visit. Because if the person is very spiritual and prayer or listening to hymns or singing [helps] with pain or anxiety, [using ENVISION would allow me to] see if maybe another visit might be something that they might appreciate sooner than later.

One chaplain user, who expressed generally positive perceptions of ENVISION’s relevance, suggested that the application would be improved by the inclusion of an indicator for “some type of spiritual distress.” With regard to the graph’s usefulness, clinician feedback was generally positive. One clinician explained as follows:

[ENVISION] would be helpful to identify patterns without having to go back and read your notes, and it would also be helpful to measure how long a pattern’s been happening when it might be hard to conceptualize that just through memory.

More general comments described ENVISION as “a really cool tool and a really good idea [that would be] really useful” and “really helpful.” Another stated as follows:

I would be eager for [ENVISION]. I think it would be great for patients’ families [to feel] like they have another method of communicating with us.

A clinician described the patient and caregiver scorecards as follows:

I think they are very helpful. It’s easy, quick to identify, and you can see exactly what the problems are.

A few clinicians specifically commented on including caregiver well-being indicators in addition to patient data, noting its usefulness:

What I really appreciate is that...it indicates an attention to continued review about how the caregiver is doing, and that isn’t always done.

A hospice physician stated as follows:

I think having access to this would really help, so I can get the patient perspective. As a hospice physician, a lot of times I’m getting just a third-party review from the nurse. I don’t necessarily get this drilled-down of a rating scale on what’s going on.

Two clinician users were more negative than positive regarding ENVISION’s purpose. One (the more ambivalent of the 2) user stated as follows:

I think it’s helpful, but is it necessary? I don’t know.

The other user explained their reservations about the application:

[My] knee-jerk response to [ENVISION] is why the heck would I be looking at a computer and not talking to [the family caregiver]? I have no idea why we would add a layer between the hospice nurse and the [patient and family]...I’m struggling with the whole concept...I’m a [age in the 60-69 range]-year-old nurse, and I’m covering two different teams...[Even] with 21 patients, I would still want to have direct conversations with my patients and families. I would want them to feel like they have no barriers whatsoever to either calling the office or calling my work cell phone and saying, “Guess what’s happening this morning?”

Data describing ENVISION’s congruence referenced the degree to which the application was aligned with users’ values, beliefs, customs, and preferences. When discussing ENVISION’s congruence, participants commented on qualities such as the application’s goodness of fit or described what they liked and disliked about it. Overall, family caregivers generally reported ENVISION to be aligned with their values, beliefs, customs, and preferences. None of the family caregivers reported perceived or anticipated challenges with daily symptom and well-being data entry. Two family caregivers mentioned specific symptoms that seemed at odds with their expectations or understanding of hospice care. One questioned why the hospice team would need to know whether they (the family caregiver) were experiencing insomnia (as previously described), and the other thought asking about patients’ lack of appetite might be problematic, as they understood decreased appetite to be a normal part of the dying process rather than something that required a clinical response: “I was just told, ‘Don’t try to make her [eat].’” Other data suggested that this family caregiver’s concern might have been warranted, as one caregiver cited “lack of appetite” as among the most important symptoms to communicate to the hospice team, explaining that a hospice patient “needs to eat.” While cultural congruence was infrequently discussed regarding the ENVISION application, the few comments provided were positive and related to cultural norms that might reduce the likelihood of unscheduled contacts with the hospice team in the absence of a tool such as ENVISION:

In an ideal world, every clinician would call [the family caregiver of a patient whose pain medications were increased] the next day to check in to see if this is working better, but I know that’s not going to be the case. A lot of caregivers actually wait a full week until the...nurse comes back, and I’m like, ‘Oh, don’t do that. Let them know that it’s working. Let them know if it’s not working. Because [patients] don’t need to suffer like that.’ There are cultural values that limit how people communicate, and that’s especially true in, like, Latino populations and other people who have been marginalized before who don’t know that they’re also an authority in this, in the reporting of patients’ symptoms.

Congruence pertaining to clinician data primarily related to clinicians’ preferences and experiences as busy professionals with limited time to engage in additional work tasks. These data primarily addressed the provision of technical support or data entry reminders to family caregivers using ENVISION, something clinicians were almost universally disinclined to take on. In addition, the previously described response from the hospice nurse with a strong preference for nontechnologically mediated communication (“Why we would add a layer between the hospice nurse and the [patient and family]?”) was identified as a likely example of perceived incongruence with the clinician’s personal values (ie, an aversion to technology or belief that more traditional forms of communication are more effective or personal). Conversely, perceptions of ENVISION as a tool to increase efficiency were strongly related to perceptions of the application as a good fit for clinicians’ workflow. For example, one clinician emphasized the timesaving value of ENVISION’s patient and caregiver scorecards:

It doesn’t seem like there’s a lot of information on [the scorecards] that doesn’t need to be there, so that’s helpful...Whenever I’m reading people’s [medical] records, I’m just like, “Where is the information I’m looking for?”

Descriptions of the application as “a quick snapshot” and as allowing clinicians to quickly identify symptoms in need of attention were common.

Data describing ENVISION’s credibility referenced the degree to which users perceived the application as trustworthy. Participants rarely commented on ENVISION’s credibility. Furthermore, 100% of the data segments labeled with the code “credibility” were also labeled with the code “safety” and were found to pertain more directly to security issues than to the perceived trustworthiness of the application. Thus, to avoid duplicate reporting of findings, these data are described in the context of ENVISION’s safety, which is discussed in the section describing findings related to ENVISION’s impact.

#### Impact

Data describing ENVISION’s benefit referenced the ways in which the application might improve users’ lives. Both family caregivers and clinicians cited potential benefits of the ENVISION application, primarily centered around better symptom management and increased awareness of opportunities to improve patients’ and family caregivers’ quality of life. Much of the information labeled with code “benefit” was also labeled with the code “relevance” due to perceived improvement in individuals’ ability to complete tasks associated with their respective roles, whether as family caregivers or hospice clinicians. For example, a hospice clinician indicated that ENVISION “would be a good communication tool [so] that...all the team is getting the same information in real time.” Another stated, “It could allow for efficient follow-up and getting the care needed to the patient probably in...a faster manner.” Several clinicians predicted that ENVISION use would increase patients’ and family caregivers’ satisfaction with the care they received. A clinician explained as follows:

It would make the patients and families feel like the hospice team is more competent, that we actually work together as a team, because we would know going in [to the home] what has been going on with them for the past few days or since we’ve been there. I do get that a lot. Patients are like, ‘I don’t want to go over it again. Don’t you guys talk to each other?’

Another clinician described ENVISION as potentially empowering:

When your patients and families are allowed to have input, it makes them feel empowered and a part of the care. I could see how [ENVISION] would be beneficial for the patients or their families to utilize.

One clinician user identified benefits from 3 perspectives:

From the caregiver’s perspective, I think it’s helpful to have some sense of feeling like you have an outlet to discuss what symptoms you’re having so that you can actually get help from the interdisciplinary team. I think it’s helpful from the patient’s perspective to kind of have a sense of control over how their symptoms are being managed...I think it’s helpful from the provider’s perspective for…symptom management, changes in medications, gauging how they’re working, as well as helping guide that family with new symptoms that are showing up and education as well as prognostication.

Comments describing potential drawbacks of the application were less frequent and often co-coded with other digital inclusivity elements. For example, clinicians worried that family caregivers with limited technological skills might feel frustrated when interacting with the application. Clinicians also worried that family caregivers would find it burdensome to enter daily symptom assessment data:

[Having daily symptom and well-being data] would be sweet. That might be a big ask for some caregivers. One more thing to do.

However, no family caregivers cited daily data entry as likely burdensome. Clinicians also cautioned against using ENVISION data to reduce or “change the care that we otherwise would attempt to provide.”

Data describing ENVISION’s safety referenced the presence or absence of protection from online threats associated with the application’s use, such as malware or data breaches. ENVISION’s safety was rarely addressed. When the users did address it, they tended to focus on password-related hassles rather than concerns that using the application made them susceptible to digital threats. One exception was that clinicians emphasized the need for any application used in hospice to be Health Insurance Portability and Accountability Act compliant, as that would likely be required for adoption into routine care.

## Discussion

### Principal Findings

We developed a conceptual framework of digital inclusivity for health information systems and then applied the framework in evaluating the digital inclusivity of ENVISION, a symptom monitoring tool for home hospice care. Our analysis identified accessibility, relevance, and impact as essential considerations in assessing a health system’s digital inclusivity; all 3 were incorporated into our newly created ARI framework. Study findings resulting from our application of the ARI framework generally supported ENVISION’s digital inclusivity, particularly concerning its perceived relevance to the work of family caregivers and hospice clinicians and its potentially positive impact on symptom management and quality of life. Limitations to ENVISION’s digital inclusivity centered around issues of accessibility, particularly operability among individuals with limited technological knowledge and skills.

The ARI framework is informed by and extends prior knowledge. It incorporates constructs from several existing models, most notably the community-oriented framework on which it was explicitly based [15]. Both frameworks place a strong emphasis on accessibility, including availability, affordability, and more standard accessibility features, conceptualized in the ARI framework as perceivability, operability, and comprehensibility (these closely mirror principles detailed in the widely referenced Web Content Accessibility Guidelines 2.0, authored by the World Wide Web Consortium) [25]. The ARI framework also echoes some of the principles highlighted in usability heuristics for user interface design given by Nielsen [26] (eg, the match between the system and the real world) and Technology Acceptance Model elaborated by Davis [27] (eg, usefulness and ease of use). In building on prior research, the ARI framework synthesizes relevant constructs from numerous bodies of existing work, setting the stage for meaningful assessment of the digital inclusivity of individual tools. In addition to providing a valuable synthesis of existing models pertinent to digital inclusivity, the ARI framework incorporates constructs uniquely relevant to the context of health information systems. It identifies patients and families, clinicians, and institutions as unique yet interdependent user types, each with potentially different cultures, responsibilities, needs, and concerns. Furthermore, it is grounded in data derived from home hospice care, a clinical context that highlights the extent to which patients and families are increasingly required to be both care providers, via family caregiving [28] and disease self-management [29], and care recipients, via patient- and family-centered models of care [30].

Importantly, the salience of specific constructs highlighted in the ARI framework will likely fluctuate over time. For example, limited internet availability may become less problematic in the United States, where the federal government’s recent infrastructure investments are predicted to significantly expand rural internet availability [31]. Similarly, while health information systems’ operability will likely continue to be important, tools requiring basic technological skills to operate may become more broadly operable due to demographic shifts, as the proportion of potential users who are digital natives (people who grew up regularly using digital technologies [32]) is expanding. Other issues, such as cost-related barriers to accessing digital health tools, seem likely to retain their importance over time, particularly in light of increased recognition of income inequality and other social determinants of health [33].

### Limitations

Study findings should be interpreted in light of numerous limitations. First, our study sample was disproportionate in terms of having higher number of non-Hispanic, White, and female individuals. All family caregivers who participated in the study had at least some college education or trade school experience, and all could speak and read English. Furthermore, while our sample reflected some variability regarding functional abilities (eg, some participants reported mild visual impairment requiring corrective lenses), no participants reported significant physical disabilities. Additional research with more diverse participants, including individuals with varying degrees of literacy and functional ability, is needed to refine the ARI framework and better understand and ultimately enhance ENVISION’s digital inclusivity. Notably, the ARI framework is in its infancy, and additional development and testing will likely be needed to maximize its potential impact on the field. In particular, noted conceptual links between relevance and the benefit subcategory of impact highlight the need for ongoing attention to issues of construct validity. With regard to ENVISION’s potential for clinical adoption, recommended next steps include pilot testing in real-world scenarios, followed by more definitive efficacy testing to determine its effect on outcomes identified by users as areas of potential impact, such as symptom management and quality of life. In addition, in developing the ARI framework, we opted to include some elements of digital inclusivity even in the absence of data from the ENVISION evaluation supporting their inclusion if existing literature or expertise among team members suggested that they were essential to the concept of digital inclusivity. For example, although participants rarely discussed safety, it was retained from the original framework in light of the large and growing number of digital security threats in existence and noted disparities in individuals’ knowledge of cybersecurity [34]. Thus, while the ARI framework is primarily grounded in data derived from our evaluation of ENVISION’s digital inclusivity, some exceptions apply. Finally, we emphasize that all study participants used ENVISION in a hypothetical manner, interacting with data either from memory (as was the case for family caregiver participants) or from fictitious patients and caregivers (as was the case for clinician participants). We cannot conclude with certainty that individuals using the application in real-life situations would have similar experiences or provide feedback mirroring that provided by the study participants. The hypothetical nature of participants’ application use also limited their ability to provide feedback on certain aspects of ENVISION, such as the application’s actual costs, including the labor and other resources required to support and sustain its use. Additional research is needed to determine ENVISION’s costs and its benefits to home hospice patients, family caregivers, and clinicians.

### Conclusions

Our evaluation of ENVISION identified many ways by which the tool is digitally inclusive. Although specific users’ experiences and feedback varied, ENVISION was determined to be generally accessible by individuals with the skills and resources required to access and operate typical web-based applications. This overall assessment was most explicitly reflected in users’ comparisons of ENVISION’s operability to that of applications that they regularly encountered in their work and personal lives. User data were most positive regarding ENVISION’s relevance, with nearly all family caregivers and clinicians readily identifying multiple use case scenarios for the application in home hospice care. Although individuals participating in the study interacted with hypothetical patient and family caregiver data, most predicted numerous, meaningful, positive outcomes of ENVISION use, including improved symptom management and patient and caregiver well-being.

User data also provided insights into ways in which ENVISION’s digital inclusivity is limited. While most Americans can access the internet from home, this capability remains limited among older adults, racial and ethnic minority groups, and individuals residing in rural communities and low-income households [35,36]. As an entirely web-based application requiring daily use, ENVISION would largely be inaccessible to individuals without high-speed internet access at home or nearby. Moreover, individuals with limited technological skills may be unable to use the application without training and support, which many hospice agencies lack the resources to provide. Minimally, our findings suggest that support would be needed to assist first-time users in logging into the system and creating a new password, as this proved to be the most challenging aspect of operating ENVISION for many users. Adoption of password alternatives (eg, biometrics, physical hardware, etc) may be considered as this technology evolves [37]. In addition, offering support in using existing tools to enhance accessibility (eg, the zoom-in feature or magnifying applications on mobile phones to enhance character visibility) may be needed. Incorporation of existing principles (eg, the usability heuristics by Nielsen) [26] into future design efforts would likely enhance operability and is thus supported by study findings. With regard to relevance, ENVISION may be a poor fit for family caregivers and clinicians who prefer face-to-face (or telephone) contact over more technologically mediated communication. Clinicians’ concerns that the application might lead to decreased face-to-face contact might be assuaged by presenting ENVISION as a tool to supplement, not substitute, in-home patient and family care. Finally, findings clearly indicate that ENVISION must provide clinicians with a net gain in terms of efficiency, consistent with existing research highlighting time constraints as the most significant professional challenge for hospice clinicians [38].

## Appendix

Multimedia Appendix 1 Original community-level digital inclusivity principles and final codes.

## Abbreviations

ARI: Accessibility, Relevance, and Impact
ENVISION: Engagement and Visualization to Improve Symptoms in Oncology Care
