# Supplementary material for: Accessibility, Relevance, and Impact of a Symptom Monitoring Tool for Home Hospice Care: Theory Elaboration and Qualitative Assessment
Source: JMIR Hum Factors. 2024 May 23;11:e51789. doi: 10.2196/51789 (PMC11157174; doi:10.2196/51789)
Supplement: Multimedia Appendix 1 [file humanfactors_v11i1e51789_app1.docx]

**Multimedia Appendix 1**. *Original community-level digital inclusivity principles and final codes*

| **Original Principle** | **Original Definition** | **Final Code** | **Final Code Definition** |
| --- | --- | --- | --- |
| **Access Principles** | Principles that address the community infrastructure needed to provide opportunities for community members to benefit from digital technologies | **Accessibility Codes** | This group of codes addresses the infrastructure, equipment, and abilities needed to access and use ENVISION. |
| - Availability | Addresses the extent to which digital technologies are available in communities | - Availability (specific codes: *availability: internet*; *availability: device*; *availability: application*) | Data reference the presence or lack of internet access, device(s) (e.g., computer, smartphone, tablet), and/or potential users’ ability to retrieve the ENVISION application (e.g., by navigating to a website or downloading an application) |
| - Affordability | Addresses the extent to which community members can afford the cost of digital technologies | - Affordability and sustainability (specific codes: *affordability and sustainability: internet*; *affordability and sustainability: device*; *affordability and sustainability: application)* | Data reference the cost of internet access, device(s) (e.g., computer, smartphone, tablet), or the ENVISION application |
| - Design for inclusion | Addresses the extent to which digital technologies are designed to ensure they are accessible to diverse groups of community members with differing abilities | - Perceivability (specific codes: *perceivability: vision*; *perceivability: hearing*) | Data reference the extent to which ENVISION can be used by individuals with different sensory abilities (e.g., visual or hearing impairments) |
|  |  | - Operability (specific codes: *operability: physical function*; *operability: technical skills*) | Data reference the extent to which ENVISION can be used by individuals with different physical abilities and/or technological proficiencies |
|  |  | - Comprehensibility (specific codes: *comprehensibility: language*; *comprehensibility: literacy*; *comprehensibility: numeracy*; *comprehensibility: visual interpretation*) | Data reference the extent to which users are able to understand and accurately interpret ENVISION content |
| - Public access | Addresses the extent to which communities have sufficient, convenient, public access to digital technologies | Not applicable | Data referencing the existence of public access to the internet or devices required to access ENVISION should be coded as *internet availability: internet* or *availability: device* (defined above). |
| **Adoption Principles** | Principles that address facilitators and barriers to purposeful use of technology, independent of whether access is available | **Relevance Codes** | This group of codes addresses the extent to which ENVISION is perceived as appropriate for its intended use/users. |
| - Relevance | Addresses the extent to which community members are aware of the potential benefits of using digital technologies | - Purpose (specific codes: *purpose: patients and families*; *purpose: clinicians*; *purpose: institution*) | Data reference ENVISION’s perceived usefulness among intended users |
|  |  | - Congruence (specific codes: *congruence: personal*; *congruence: cultural*; *congruence: institutional*) | Data reference the degree to which ENVISION is aligned with users’ values, beliefs, customs, and preferences |
|  |  | - Credibility (specific codes: *credibility: patients and families*; *credibility: clinicians*; *credibility: institution*) | Data reference the degree to which ENVISION is perceived as trustworthy |
| - Digital literacy | Addresses the extent to which community members possess the knowledge and skills necessary to find, evaluate, and use information to achieve goals | Relocated (see above) | Data referencing digital literacy skills required to use ENVISION should be coded as *operability: technical skills* (defined above)*.* |
| - Consumer safety | Addresses the extent to which communities support safe navigation of the digital world | Relocated (see below) | Data referencing users’ safety should be coded as *safety: patients and families*, *safety: clinicians*, or *safety: institution* (defined below). |
| **Application Principles** | Principles that address specific purpose areas where thoughtful deployment of technologies could benefit community wellbeing | **Impact Codes** | This groups of codes addresses the potential outcomes of ENVISION use. |
| - Health care | Addresses the potential for digitally inclusive communities to benefit with regard to health care | - Benefit (specific codes: *benefit: patients and families*; *benefit: clinicians*; *benefit: institution*) | Data reference ways ENVISION improves (or would be expected to improve) users’ lives |
|  |  | - Safety (specific codes: *safety: patients and families*; *safety: clinicians*; *safety: institution*) | Data reference the presence or absence of protection from online threats associated with ENVISION use |
| - Economic and workforce development | Addresses the potential for digitally inclusive communities to benefit with regard to economic and workforce development | Not applicable | Not applicable |
| - Education | Addresses the potential for digitally inclusive communities to benefit with regard to education | Not applicable | Not applicable |
| - Public safety and emergency services | Addresses the potential for digitally inclusive communities to benefit with regard to public safety and emergency services | Not applicable | Not applicable |
| - Civic engagement | Addresses the potential for digitally inclusive communities to benefit with regard to civic engagement | Not applicable | Not applicable |
| - Social connections | Addresses the potential for digitally inclusive communities to benefit with regard to social connections | Not applicable | Not applicable |
